# Supplementary material for: Arsenicals, the Integrated Stress Response, and Epstein–Barr Virus Lytic Gene Expression
Source: Viruses. 2021 Apr 30;13(5):812. doi: 10.3390/v13050812 (PMC8147158; doi:10.3390/v13050812)
Supplement: Supplementary file 1 [file viruses-13-00812-s001.zip › viruses-1169506-supplementary.pdf]

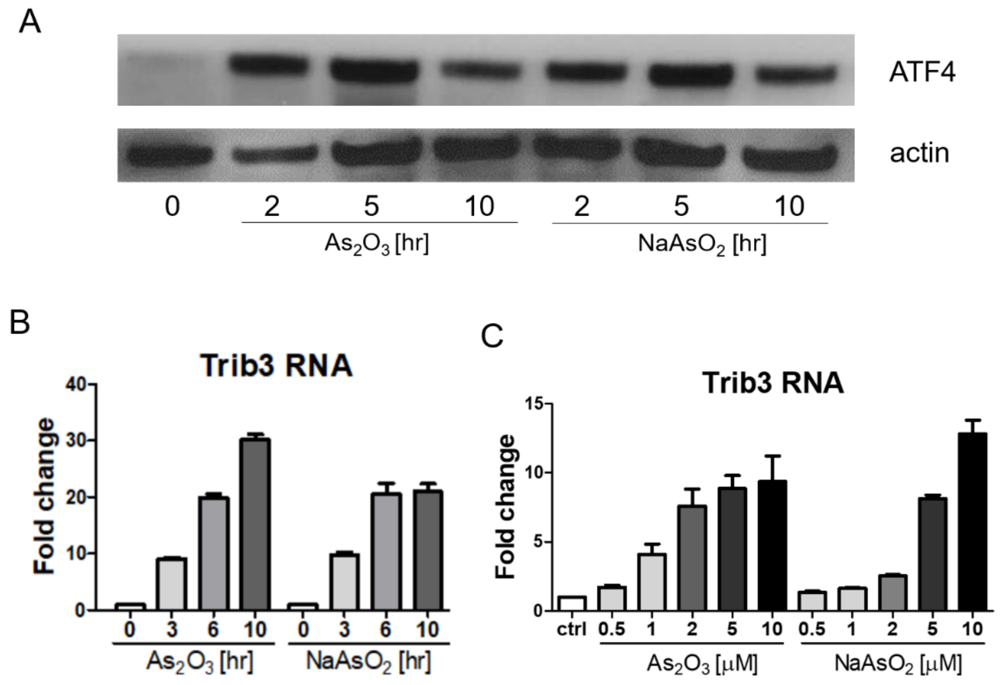

Figure S1. Arsenic leads to ATF4 and Trib3 expression. (A) BX1-Akata cells were treated with 10μM As_2O_3 or $NaAsO_2$ for indicated time period and expression of ATF4 proteins were measured by western blot. (B) BX1-Akata cells were treated with 10μM As_2O_3 or $NaAsO_2$ for indicated time period and isolated RNA were used to perform qRT-PCR for detecting Trib3 RNA expression. (C) BX1-Akata cells were treated with indicated doses of As_2O_3 or $NaAsO_2$ for 24 hours and isolated RNA were used to perform qRT-PCR detecting Trib3 RNA level.

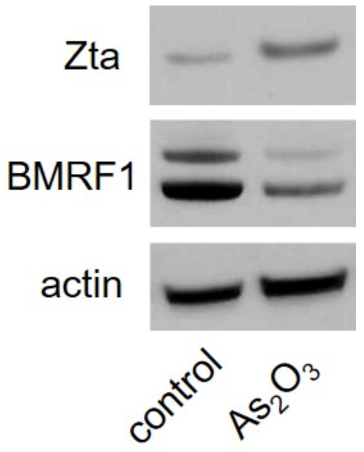

Figure S2. Arsenic trioxide increases Zta but decreases BMRF1 expression. Akata cells were treated with 10μM As_2O_3 for 24 hours and expression of proteins were measured by western blot.
